# The Complete Nucleotide Sequence of the Mitochondrial Genome of *Bactrocera minax* (Diptera: Tephritidae)

**DOI:** 10.1371/journal.pone.0100558

**Published:** 2014-06-25

**Authors:** Bin Zhang, Francesco Nardi, Helen Hull-Sanders, Xuanwu Wan, Yinghong Liu

**Affiliations:** 1 Key Lab of Integrated Pest Management of Shandong Province, College of Agronomy and Plant Protection, Qingdao Agricultural University, Qingdao, China; 2 Chongqing Key Laboratory of Entomology and Pest Control Engineering, College of Plant Protection, Southwest University, Chongqing, China; 3 Sichuan Plant Protection Station, Chengdu, China; 4 Department of Evolutionary Biology, University of Siena, Siena, Italy; 5 Department of Entomology, Pennsylvania State University, University Park, Pennsylvania, United States of America; International Atomic Energy Agency, Austria

## Abstract

The complete 16,043 bp mitochondrial genome (mitogenome) of *Bactrocera minax* (Diptera: Tephritidae) has been sequenced. The genome encodes 37 genes usually found in insect mitogenomes. The mitogenome information for *B. minax* was compared to the homologous sequences of *Bactrocera oleae*, *Bactrocera tryoni*, *Bactrocera philippinensis*, *Bactrocera carambolae*, *Bactrocera papayae*, *Bactrocera dorsalis*, *Bactrocera correcta*, *Bactrocera cucurbitae* and *Ceratitis capitata*. The analysis indicated the structure and organization are typical of, and similar to, the nine closely related species mentioned above, although it contains the lowest genome-wide A+T content (67.3%). Four short intergenic spacers with a high degree of conservation among the nine tephritid species mentioned above and *B. minax* were observed, which also have clear counterparts in the control regions (CRs). Correlation analysis among these ten tephritid species revealed close positive correlation between the A+T content of zero-fold degenerate sites (P_0FD_), the ratio of nucleotide substitution frequency at P_0FD_ sites to all degenerate sites (zero-fold degenerate sites, two-fold degenerate sites and four-fold degenerate sites) and amino acid sequence distance (ASD) were found. Further, significant positive correlation was observed between the A+T content of four-fold degenerate sites (P_4FD_) and the ratio of nucleotide substitution frequency at P_4FD_ sites to all degenerate sites; however, we found significant negative correlation between ASD and the A+T content of P_4FD,_ and the ratio of nucleotide substitution frequency at P_4FD_ sites to all degenerate sites. A higher nucleotide substitution frequency at non-synonymous sites compared to synonymous sites was observed in *nad4*, the first time that has been observed in an insect mitogenome. A poly(T) stretch at the 5′ end of the CR followed by a [TA(A)]*_n_*-like stretch was also found. In addition, a highly conserved G+A-rich sequence block was observed in front of the poly(T) stretch among the ten tephritid species and two tandem repeats were present in the CR.

## Introduction

The family Tephritidae, generally known as “true” fruit flies, includes 471 genera and 4257 species distributed throughout the temperate and tropical areas of the world. Many species are of critical importance to man either as pests of fruit and vegetable crops or as beneficial species for the control of weeds [1]. The fruit fly *Bactrocera minax* Enderlein (Diptera: Tephritidae), generally known as the Chinese citrus fruit fly, has been a serious pest of commercial citrus crops in China for more than half a century [2]. This species has been recorded in southern China, India (West Bengal and Sikkim) and Bhutan [2], [3] wild and cultivated citrus species [4]. Some hosts are endemic to southern China and the eastern Himalayan region [5] but *B. minax* has been reported on the kumquat *Fortunella crassifolia* [6] and the boxthorn *Lycium chinense* [2].

*B. minax* was first collected from India and Sikkim and designated *B. minax* Enderlein [1]. Drew [3] provided a detailed description and illustration of the *B. minax* type specimens collected in 1920 and assigned the species to the genus *Bactrocera* (*Polistomimetes*). White and Wang [7] designated a lectotype of *B. minax* and assigned the species to the *Bactrocera* (*Tetradacus*); in addition, they indicated that *Bactrocera citri* Chen, collected from China in 1940, should be placed in synonymy with *B. minax*.

A wide variety of questions about the biology and phylogeny of *B. minax* have been addressed with the aid of molecular tools. These studies could have used two main sources of genetic data; namely, nuclear sequence data and, most frequently, mitochondrial sequence data. Insect mitochondrial DNA (mtDNA) usually occurs as a double-stranded closed circular molecule, ranging in size from 14–20 kb and generally encoding 13 protein-coding genes (PCGs), two ribosomal RNAs (rRNAs) and 22 transfer RNA (tRNAs), which is conserved across bilaterian metazoans with only a few exceptions (e.g. loss of a small number of genes in some derived groups) [8]. The molecule contains at least one sequence of variable length known as the A+T-rich region or control region (CR), which contains initiation sites for transcription and replication [9] and ranges in size from tens to several thousands of base pairs [10]–[13]. As the results of highly conservative gene structures among phyla, maternal inheritance, high copy number and relatively fast evolution rates compared to nuclear DNA [14], mitochondrial genome (mitogenome) sequences have been regarded as useful molecular markers in studies focusing on comparative and evolutionary genomics, molecular evolution, phylogenetics, phylogeography and population genetics [15].

Many complete or nearly complete mitogenomes have been sequenced and comparative analyses at the genus or species level have used multiple complete mitochondrial genes instead of one or partial genes, including molecular systematics [16]–[20], population genetics/phylogeography [16], diagnostics [21], molecular evolutionary studies [13], [22], [23], the frequency and type of gene rearrangements [24], [25] and the evolution of genome size [26]. To date, more than 500 insect mitogenomes have been sequenced from all orders, including 77 dipterans in 24 families, and are available in Genbank. In this study, we sequenced the complete sequence of the mitogenome of *B. minax* (Diptera: Tephritidae).

Genbank contains information for only ten Tephritidae species; *Bactrocera oleae*, *Bactrocera tryoni*, *Bactrocera philippinensis*, *Bactrocera carambolae*, *Bactrocera papayae*, *Bactrocera dorsalis*, *Bactrocera correcta*, *Bactrocera cucurbitae*, *Ceratitis capitata* and *B. minax*. Nine of these species belong to the genus *Bactrocera*, including four species of the *B. dorsalis* species complex; the other species belongs to the genus *Ceratitis*. Within the nine *Bactrocera* species, *B. philippinensis*, *B. carambolae*, *B. papayae* and *B. dorsalis* belong to the *B. dorsalis* species complex, *B. correcta*, *B. cucurbitae* and *B. tryoni* belong to other species-groups within the subgenus *Bactrocera*, and *B*. *oleae* and *B. minax* belong to the subgenus *Daculus* and *Tetradacus*, respectively. Although recent molecular evidence suggests *B. papaya*, *B. philippinensis* and *B. dorsalis* likely represent one species [27]–[30], with anticipation of the analysis of the *B. minax* mitogenome, we compare the sequence and mitogenome origins to the tephritid species *B*. *oleae*, *B. dorsalis*, *B. philippinensis*, *B. carambolae*, *B. papayae*, *B. correcta*, *B. cucurbitae B. tryoni* and *C*. *capitata*.

## Materials and Methods

### 1. Insect and mtDNA extraction, protein-coding genes and sequencing

**Figure 1:**
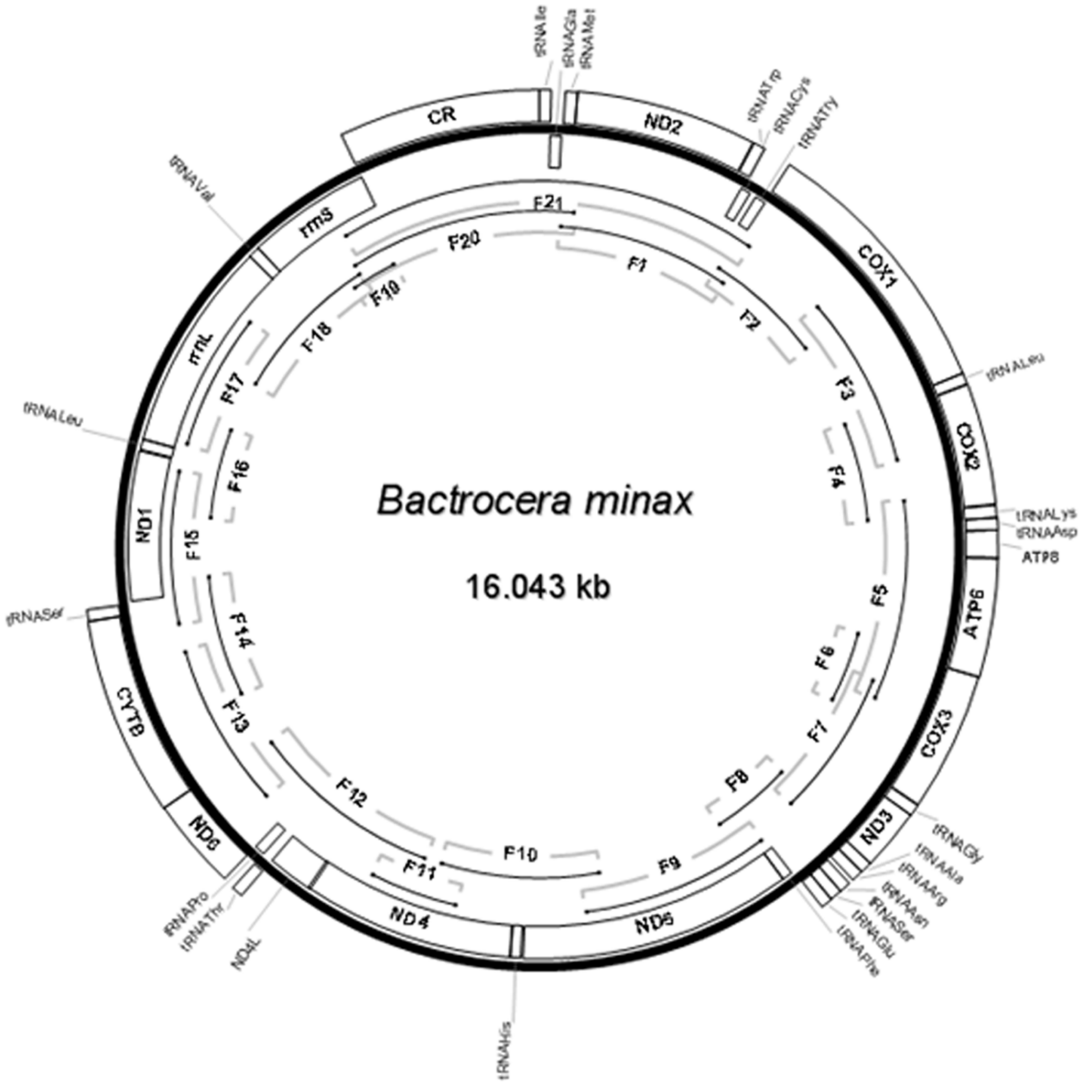
Circular map of the mitogenome of *B. minax*. The genes located outside adjoined the bold line circle (J-strand) indicated that the direction of transcription is opposite to the genes located inside adjoined the bold line circle (N-strand). *B. minax* complete mitogenome was jointed using 21 (F1–F21) fragments shown as single lines within the bold line circle.

We collected *B. minax* adults from a citrus garden on private land at Xianli Zeng covering an area of 20 hectares in Wulong (Chongqing Province, China). We confirm that Mr Zeng, the owner of this land, allowed us to conduct the study on this site. No specific permission was required for this location and our activity. We confirm the field studies did not involve endangered or protected species. *B. minax* adults were stored at 25°C in 99% (v/v) ethanol. Morphological identification was done according to White and Wang [7]. Total DNA was isolated from three adult specimens using the DNeasy Blood & Tissue kit (QIAGEN) according to the manufacturer's instructions. The whole *B. minax* mitogenome sequence was assembled from a single individual (three repeats). Purified total DNA was used as a template for amplification of the entire *B. minax* mitogenome in 21 overlapping pieces, ranging in size from 388 bp to 1762 bp. PCR primers were designed as described [31] and by comparison to the available sequences of *B*. *oleae*, *B. dorsalis*, *B. philippinensis*, *B. carambolae*, *B. papayae*, *B. correcta*, *B. cucurbitae B. tryoni* and *C*. *capitata* (Table 1). Amplification was done in a thermocycler (Eppendorf Mastercycler 5333) in 50 µl reactions containing 5 µl of 25 mM MgCl_2_, 5 µl of 10×PCR Buffer (Mg^2+^ free), 8 µl of a dNTP mixture (2.5 mM each), 3 µl of 10 µM each primer, 0.5 µl of 5 U/µl *Taq* polymerase (Takara Biomedical, Japan) and 2 µl of a 1/10 dilution of the DNA extract. Amplification conditions were: 5′ of pre-PCR denaturation at 94°C followed by 34 cycles of 30 s at 94°C, 1 min at 40–58°C (depending on the primer pair) and 2 min at 72°C. The F21 fragment (Fig. 1) was amplified using LA *Taq* (Takara Biomedical, Japan) and a cycle consisting of a pre-PCR denaturation at 96°C for 2 min followed by 30 cycles of 10 s at 98°C and 2 min at 58°C with a final elongation step of 10 min at 72°C. PCR products were separated by electrophoresis and purified using a QIAquick Gel Extraction Kit (QIAGEN). PCR products were sequenced directly on both strands using amplification and additional *ad hoc* primers as needed. Individual sequences were combined in a consensus contig using DNAStar package software (DNAStar Inc.).

**Table 1:** Summary of primers used for complete mtgenome of *B. minax* amplification.

Fragment	Upper primer	Sequence	Location	Down primer	Sequence	Location	Fragment Length
F1	F1-Ur	GCTAATTAAGCTACTGGGTTCAT	155–177	F1-Dr	TGTTCCTACTATTCCGGCTCA	1539–1560	1406
F2	F2-Ur	TACAATCTATCGCCTAAACTTCAGCC	1442–1468	F2-Ds	TAGGCACGAGTATCTACATCTAT	2357–2379	938
F3	F3-Us	GATTCTTTGGACACCCAGAA	2175–2194	F3-Ds	ATTCATAACTTCAATATCATTG	3383–3406	1232
F4	F4-Ur	ATGGCAGATTAGTGCAATGG	3016–3035	F4-Dr	GTTTAAGAGACCAGTACTTG	3789–3810	795
F5	F5-Ur	GAAATTTGCGGGGCTAATCATAG	3670–3692	F5-Dr	GAGGTCATATAGCTCCCAGTTCAAT	5072–5096	1427
F6	F6-Ur	ATCAGCTGTTGCTATTATTCA	4670–4690	F6-Dr	ACTGTAAAAAATAACCCTTGTG	5223–5245	576
F7	F7-Ur	GTAACATTAGGATAACGGTGAGGAA	4967–5991	F7-Ds	TGCAATAAATCGCTTCATATTCT	6027–6049	1083
F8	F8-Us	TATCGGCCTATACCAGGAAGGA	5908–5929	F8-Dr	GATCAAGGTTGGTCAGAA	6543–6560	653
F9	F9-Ur	AATTACCCTAACATCTTCAGTG	6355–6376	F9-Ds	TATCTAATCGGATTGGAGATGT	7684–7705	1351
F10	F10-Ur	GCTCTCTTAGTTATAGCTGC	7546–7565	F10-Ds	GGTAAGCATTAGTCTGGTT	8783–8801	1256
F11	F11-Us	ACAAAACAAACCTGACGAAC	8600–8619	F11-Ds	TAGTAGAATGAATCTTTTTATA	9215–9236	637
F12	F12-Us	GGGGCCTCAACATGAGCCCT	8913–8932	F12-Ds	TTTACAACTGCGATTAGGGT	10422–10441	1529
F13	F13-Us	AGGAGGTATATTAGTTCTATTCA	10139–10161	F13-Ds	GCAAATAGGAAGTATCATTC	11297–11314	1176
F14	F14-Us	AGCAACAGCATTCATAGGATA	10858–10878	F14-Ds	CTTTACCTCGTTTTCGTTATGAT	11802–11824	967
F15	F15-Ur	ACATGAATTGGAGCACGACCAGT	11492–11511	F15-Dr	GTGGCTTTTTTAACTCTTTTGGAACG	12556–12579	1088
F16	F16-Ur	TAGAATTAGAAGATCAGCCAGC	12254–12275	F16-Ds	ACTTTAGGGATAACAGCGTA	12960–12979	726
F17	F17-Us	TTCTAATACCTGGTCCTTTC	12757–12776	F17-Ds	CGTTTATTAGGGTATCTGGTTT	13713–13736	980
F18	F18-Ur	ATGTTTTTGTTAAACAGGCG	13360–13379	F18-Dr	AGACTAGGATTAGATACCCTATTAT	14555–14574	1215
F19	F19-Us	TACAGGACAGGTTCCTCTG	14458–14476	F19-Ds	GCGTGTATTTTTGCTTATTTA	14826–14845	388
F20	F20-Ur	AGGGTATCTAATCCTAGTTT	14557–14576	F20-Dr	AGTGATTAGGGTTCCTGTTATTA	254–275	1762
F21	F21-Us	ACTCCTACTACTTTAGCGTT	14618–14637	F1-Dr	TGTTCCTACTATTCCGGCTCA	1539–1560	2986

Note: Lowercase “r” behind some primer names represents these primers were designed on basis of Simon et al. (1994); Lowercase “r” behind some primer names represents these primers were designed by us.

### 2. Sequence analysis and gene annotation

Genes encoded on the *B. minax* mitogenome were located initially by comparison to homologous full-length insect mitochondrial sequences using DNAStar. Nucleotide sequences of PCGs were translated using the invertebrate mtDNA genetic code. tRNA genes were identified initially using tRNAscan-SE Search Server version 1.21 (available online at http://lowelab.ucsc.edu/tRNAscan-SE/) [32] and refined using tRNAscan-SE and RNAshapes [33]. The presence and secondary structures of tRNA genes that could not be located by tRNAscan-SE owing to variant morphology were annotated manually by comparison to the sequences of other insect tRNAs [34]–[37]. Codon usage analysis and relative synonymous codon usage (RSCU) in PCGs were calculated using CodonW version 1.4.2 (John Peden, available at http://codonw.sourceforge.net/index.html) [38]. Potential secondary structure folds of non-coding sequences and sequences in the CR were calculated with the DNA mfold web server using default settings (http://mfold.bioinfo.rpi.edu/cgi-bin/dna-form1.cgi) [39]. The presence of tandem repeats in the CR was investigated using the Tandem Repeats Finder available online (http://tandem.bu.edu/trf/trf.html) [40]. The A+T content and nucleotide substitution frequency at synonymous sites and non-synonymous sites (the number of synonymous substitutions per site and the number of non-synonymous substitutions per site) were calculated on the basis of the data using MEGA 4.0 [41]. The correlation analysis was done by the bivariate method using SPSS version 13 (SPSS Inc., Chicago, IL). The overall average amino acid distance among each of the PCGs from ten tephritid species (*B. minax*, *B*. *oleae*, *B. tryoni B. dorsalis B. philippinensis*, *B. carambolae*, *B. papayae*, *B. correcta*, *B. cucurbitae* and *C*. *capitata*) were calculated by the method of Poisson distances by MEGA 4.0 [41]. The complete *B. minax* mtDNA sequence was deposited in Genbank under accession no. HM776033.

## Results and Discussion

### 1. Genome organization

The mitochondrial genome of *B. minax* is a closed circular molecule of 16043 bp; hence, it is longer than the other nine tephritid mitogenomes available (range 15,815 bp in *B. oleae* to 15,980 bp in *C*. *capitata*) but is still well within the range of other insect mitogenomes (14,503 bp in *Rhopalomyia pomum* [42] to 19517 bp in *Drosophila melanogaster* [11]). The gene content is typical of metazoan mitogenomes, with 13 PCGs (*cox1*-*3*, *cob*, *nad1*-*6*, *nad4l*, *atp6* and *atp8*), 22 tRNAs and two genes for ribosomal RNA subunits (*rrnS* and *rrnL*). A long uninterrupted non-coding region of 1141 bp, likely homologous to the insect A+T-rich region, is present between *rrnS* and *trnI*, corresponding to position 14,903 to 16,043 in the annotated sequence. The gene order in the *B. minax* mitogenome corresponds to the typical and plesiomorphic state hypothesized for the Pancrustacea, and is shared with all tephritids analyzed to date (Fig. 1).

Genes in the *B. minax* mitogenome overlap by a total of 43 bp, distributed in 12 segments from 1 to 17 bp long and are separated by a total of 178 bp dispersed in 16 intergenic spacers from 2 to 42 bp (without taking the tRNA-like sequence into account; Table 2). Despite its relatively large size, the *B. minax* mitogenome has more overlapping sequences between genes compared to those of other tephritids; genes overlap by a total of 35 bp at 11 boundaries in *B. oleae*, 29 bp in seven locations in *B. tryoni*, 27 bp in five locations in *B. dorsalis*, 34 bp in ten locations in *B. philippinensis*, 32 bp in nine locations in *B. carambolae*, 34 bp in ten locations in *B. papayae*, 35 bp in 11 locations in *B. correcta*, 32 bp in nine locations in *B. cucurbitae* and only 3 bp at three boundaries in *C. capitata*.

**Table 2:** Summary of *B. minax* mitogenome.

Gene	Direction	Location	Size	IGS	Anticodon	Start code	Stop code
*trnI*	F	1–65	65	0	GAT		
*trnQ*	R	66–134	69	10	TTG		
*trnM*	F	145–213	69	−1	CAT		
*nad2*	F	213–1235	1023	8		ATT	TAG
*trnW*	F	1244–1311	68	−8	TCA		
*trnC*	R	1304–1365	62	42	GCA		
*trnY*	R	1408–1475	68	−2	GTA		
*cox1*	F	1474–3009	1536	−1		TCG	TAT
*trnL^(UUR)^*	F	3009–3072	65	5	TAA		
*cox2*	F	3078–3764	687	6		ATG	TAA
*trnK*	F	3771–3841	71	−1	CTT		
*trnD*	F	3841–3908	68	0	GTC		
*atp8*	F	3909–4070	162	−7		ATT	TAA
*atp6*	F	4064–4741	678	−1		ATG	TAG
*cox3*	F	4741–5532	792	6		ATG	TAA
*trnG*	F	5539–5604	66	0	TCC		
*nad3*	F	5605–5956	352	0		GTC	T
*trnA*	F	5957–6021	65	5	TGC		
*trnR*	F	6027–6090	64	28	TCG		
*trnN*	F	6119–6183	65	0	GTT		
*trnS^(AGN)^*	F	6184–6251	68	2	GCT		
*trnE*	F	6254–6319	66	18	TTC		
*trnF*	R	6338–6403	66	−1	GAA		
*nad5*	R	6403–8122	1720	14		ATT	T
*trnH*	R	8137–8201	65	4	GTG		
*nad4*	R	8206–9546	1341	−17		ATG	TAA
*nad4l*	R	9530–9826	297	2		ATG	TAA
*trnT*	F	9829–9893	65	0	TGT		
*trnP*	R	9894–9959	66	2	TGG		
*nad6*	F	9962–10483	522	−1		ATG	TAA
*cob*	F	10483–11619	1137	−2		ATG	TAG
*trnS^(UCN)^*	F	11618–11684	67	16	TGA		
*nad1*	R	11701–12640	940	10		ATA	T
*trnL^(CUN)^*	R	12651–12716	66	0	TAG		
*rrnL*	R	12717–14049	1333	−1			
*trnV*	R	14049–14120	72	0	TAC		
*rrnS*	R	14121–14902	782	0			
*CR*		14903–16043	1141	0			

### 2. Nucleotide composition

The overall base composition of *B. minax* is 38.0% A, 11.2% G, 29.3% T and 21.5% C. Similar to other insect sequences, the *B. minax* mitogenome nucleotide composition is biased toward adenine and thymine (67.3% A+T), which is the lowest value among the tephritid mitogenomes available. Analyzed separately, all PCGs (64.3%), tRNAs (72.2%), sRNAs (73.7%) and CR (77.6%) have the lowest A+T content compared to the other known tephritid mitogenomes (Table 3).

**Table 3:** Length and base composition of different genomic regions in 10 tephritid species, *B. oleae*, *B. tryoni*, *B. philippinensis*, *B. carambolae*, *B. papaya*, *B. dorsalis*, *C. capitata*, *B. minax*, *B. correcta* and *B. curcubitae*.

Accession No. and speices	Whole mtDNA		PCGs		tRNAs		rRNAs		CR	
	Size	(A+T)%	Size	(A+T)%	Size	(A+T)%	Size	(A+T)%	Size	(A+T)%
AY210702 *B. oleae*	15815	72.6	11188	70.1	1484	75.1	2116	77.1	949	86.9
HQ130030 *B. tryoni*	15925	72.5	11186	69.6	1467	75.0	2115	77.7	951	87.0
DQ995281 *B. philippinensis*	15915	73.6	11192	71.1	1466	75.3	2114	77.7	949	88.2
EF014414 *B. carambolae*	15915	73.6	11190	71.2	1466	75.1	2113	77.6	950	87.9
DQ917578 *B. papayae*	15915	73.5	11190	71.0	1465	75.1	2114	77.7	950	88.2
DQ 845759 *B. dorsalis*	15915	73.6	11185	71.2	1467	75.2	2123	77.8	949	88.1
AJ242872 *C. capitata*	15980	77.5	11272	75.5	1472	76.8	2123	80.2	1004	91.1
HM776033 *B. minax*	16043	67.3	11187	64.3	1466	72.2	2115	73.7	1141	77.6
JX456552 *B. correcta*	15936	73.2	11192	71.2	1470	75.3	2117	77.9	949	78.6
JN635562 *B. curcubitae*	15825	72.8	11190	70.7	1467	75.1	2110	77.8	946	82.3

Considering the two strands separately, the PCGs on the Majority strand (J-strand, nine PCGs are located on this strand) (61.5%) have a lower A+T content compared to the Minority strand (N-strand, the other four PCGs are located on this strand) (68.9%). Furthermore, PCGs encoded on the J-strand have a comparable content of A (31.0%) and T (30.5%), whereas PCGs on the N-strand show a strong bias for T content (46.3%) compared to A content (22.6% A). The above situation has been observed in the other tephritid mitogenomes available (data not shown) and in other insects [34]–[37], [43]–[50]. However, tRNAs on the two opposite strands have nearly equal A+T contents, which has been found in the other nine tephritid species. For three PCG codon positions, the third codon positions have significantly higher A+T content than the first and second codon positions owing to genetic code degeneracies. In particular, T in each codon position of PCGs on the N-strand is over-represented. With exception of the second codon position over-representing T, however, the first and third codon positions of PCGs show a preponderance of A on the J-strand and T on the N-strand, which is similar to many insect mitogenomes [34]–[37], [43]–[50] (Table 3).

The base compositional bias for A+T in PCGs is reflected in the relative synonymous codon usage statistics of the *B. minax* mitogenome (Table 4). With the exception of amino acid His, codons with A or T in the third codon position are generally strongly over-represented compared to codons terminating with either G or C. The ratio of G+C-rich (Pro, Ala, Arg and Gly) codons to A+T-rich codons (Phe, Ile, Met, Tyr, Asn and Lys) in *B. minax* PCGs was 0.44, which is higher compared to the other nine tephritids *B. dorsalis* (0.29), *B. philippinensis* (0.29), *B. carambolae* (0.30), *B. papayae* (0.29), *B. correcta* (0.30), *B. cucurbitae* (0.32), *B. oleae* (0.31), *B. tryoni* (0.32) and *C. capitata* (0.23). This demonstrates the amino acid composition is affected by the lower A+T mutational bias in *B. minax* (67.3%) and the stronger A+T mutational bias in *B. dorsalis* (73.6%), *B. philippinensis* (73.6%), *B. carambolae* (73.6%), *B. papayae* (73.5%), *B. correcta* (73.2%), *B. cucurbitae* (72.8%), *B. oleae* (72.6%), *B. tryoni* (72.4%) and *C. capitata* (77.5%).

**Table 4:** Relative synonymous codon usage of 10 tephritid species, 10 tephritid species, *B. oleae*, *B. tryoni*, *B. philippinensis*, *B. carambolae*, *B. papaya*, *B. dorsalis*, *C. capitata*, *B. minax*, *B. correcta* and *B. curcubitae*.

Amino acid	Codon	*B. minax*			*B. dorsalis*			*B*. *oleae*			*B. tryoni*			*C*. *capitata*			*B. philippinensis*			*B. carambolae*			*B. papaya*			*B. correcta*			*B. curcubitae*		
		All	J	N	All	J	N	All	J	N	All	J	N	All	J	N	All	J	N	All	J	N	All	J	N	All	J	N	All	J	N
Phe	UUU	**1.37**	**1.06**	**1.69**	**1.52**	**1.37**	**1.73**	**1.48**	**1.25**	**1.77**	**1.44**	**1.31**	**1.72**	**1.68**	**1.67**	**1.79**	**1.51**	**1.36**	**1.73**	**1.50**	**1.35**	**1.72**	**1.51**	**1.36**	**1.73**	**1.48**	**1.39**	**1.81**	**1.46**	**1.34**	**1.69**
	UUC	0.63	0.94	0.31	0.48	0.63	0.27	0.52	0.75	0.23	0.56	0.69	0.28	0.32	0.33	0.21	0.49	0.64	0.27	0.50	0.65	0.28	0.49	0.64	0.27	0.52	0.61	0.19	0.54	0.66	0.31
Leu	UUA	**2.11**	1.41	**2.52**	**2.76**	**2.44**	**3.27**	**2.70**	**2.44**	**3.07**	**2.50**	**2.07**	**2.94**	**3.62**	**4.00**	**4.04**	**2.78**	**2.51**	**3.25**	**2.84**	**2.52**	**3.28**	**2.79**	**2.52**	**3.27**	**2.70**	**2.29**	**3.42**	**2.93**	**2.68**	**3.20**
	UUG	1.09	0.38	2.03	0.84	0.30	1.33	0.85	0.31	1.60	0.89	0.24	1.44	0.74	0.36	0.88	0.81	0.30	1.28	0.86	0.33	1.36	0.86	0.33	1.37	0.67	0.31	0.89	0.78	0.46	1.32
	CUU	0.98	1.23	0.72	1.10	1.59	0.77	0.98	1.14	0.81	1.08	1.62	0.79	0.87	0.87	0.70	1.08	1.56	0.77	1.11	1.59	0.77	1.07	1.53	0.70	1.03	1.48	0.72	0.98	1.49	0.68
	CUC	0.46	0.83	0.18	0.21	0.30	0.08	0.35	0.48	0.05	0.31	0.38	0.06	0.13	0.15	0.03	0.19	0.26	0.08	0.16	0.23	0.08	0.19	0.24	0.11	0.30	0.34	0.25	0.26	0.25	0.12
	CUA	0.96	**1.81**	0.36	0.79	1.15	0.40	0.89	1.40	0.34	0.94	1.33	0.53	0.44	0.54	0.21	0.80	1.12	0.43	0.73	1.07	0.32	0.77	1.13	0.38	1.03	1.30	0.58	0.84	0.98	0.62
	CUG	0.39	0.36	0.18	0.30	0.23	0.16	0.23	0.22	0.13	0.28	0.36	0.24	0.19	0.08	0.13	0.34	0.26	0.19	0.31	0.26	0.19	0.32	0.26	0.19	0.29	0.27	0.14	0.21	0.14	0.06
Ile	AUU	**1.42**	**1.23**	**1.83**	**1.72**	**1.66**	**1.84**	**1.56**	**1.50**	**1.87**	**1.59**	**1.61**	**1.77**	**1.77**	**1.72**	**1.83**	**1.71**	**1.67**	**1.83**	**1.73**	**1.69**	**1.85**	**1.70**	**1.64**	**1.81**	**1.70**	**1.65**	**1.83**	**1.52**	**1.51**	**1.61**
	AUC	0.58	0.77	0.17	0.28	0.34	0.16	0.44	0.50	0.13	0.41	0.39	0.23	0.23	0.28	0.17	0.29	0.33	0.17	0.27	0.31	0.15	0.30	0.36	0.19	0.30	0.35	0.17	0.48	0.49	0.39
Met	AUA	**1.33**	**1.45**	**1.20**	**1.37**	**1.46**	**1.42**	**1.41**	**1.46**	**1.37**	**1.43**	**1.43**	**1.51**	**1.53**	**1.44**	**1.69**	**1.38**	**1.47**	**1.45**	**1.40**	**1.44**	**1.46**	**1.38**	**1.45**	**1.41**	**1.49**	**1.43**	**1.52**	**1.48**	**1.40**	**1.56**
	AUG	0.67	0.55	0.80	0.63	0.54	0.58	0.59	0.54	0.63	0.57	0.57	0.49	0.47	0.56	0.31	0.62	0.53	0.55	0.60	0.56	0.54	0.62	0.55	0.59	0.51	0.57	0.48	0.52	0.60	0.44
Val	GUU	**1.46**	0.63	**2.09**	**1.52**	1.14	**2.04**	1.41	0.74	**2.29**	**1.55**	1.32	**1.94**	**2.31**	**2.10**	**2.50**	**1.59**	1.19	**2.11**	**1.64**	1.23	**2.15**	**1.63**	1.28	**2.11**	**1.75**	1.47	**2.20**	**1.67**	1.43	**2.03**
	GUC	0.48	0.77	0.18	0.44	0.42	0.30	0.39	0.63	0.25	0.52	0.49	0.56	0.27	0.07	0.39	0.41	0.42	0.30	0.34	0.35	0.31	0.38	0.36	0.30	0.24	0.42	0.12	0.46	0.35	0.42
	GUA	1.25	**1.88**	0.93	1.49	**2.23**	1.09	**1.70**	**2.40**	0.96	1.42	**1.71**	1.06	1.13	1.63	0.83	1.41	**2.13**	0.99	1.45	**2.22**	0.97	1.39	**2.15**	0.95	1.56	**1.84**	1.22	1.59	**2.07**	1.13
	GUG	0.81	0.72	0.81	0.55	0.21	0.57	0.51	0.23	0.50	0.52	0.49	0.44	0.30	0.20	0.28	0.59	0.26	0.60	0.57	0.20	0.57	0.60	0.21	0.65	0.45	0.26	0.46	0.28	0.15	0.42
Ser	UCU	1.29	0.96	**2.19**	1.66	1.20	**2.13**	**1.61**	1.43	**1.97**	**2.14**	1.48	**2.29**	**2.02**	1.59	**2.06**	1.67	1.20	**2.13**	1.62	1.13	**2.16**	1.70	1.21	**2.13**	**1.94**	1.28	**2.59**	**1.92**	1.50	**1.99**
	UCC	1.17	1.14	0.31	0.65	0.63	0.35	0.85	0.76	0.36	0.48	0.43	0.34	0.56	0.74	0.24	0.63	0.63	0.40	0.64	0.63	0.35	0.59	0.55	0.35	0.62	0.59	0.29	0.69	0.72	0.53
	UCA	**1.35**	**1.26**	0.66	**1.81**	**1.93**	0.86	1.48	**1.53**	1.07	1.68	**1.88**	0.95	1.67	**1.69**	1.24	**1.74**	**1.88**	0.86	**1.81**	**1.95**	0.82	**1.74**	**1.89**	0.86	1.68	**1.84**	1.00	1.57	**1.57**	1.11
	UCG	0.53	0.29	0.31	0.46	0.31	0.23	0.46	0.28	0.36	0.40	0.40	0.22	0.28	0.23	0.12	0.44	0.29	0.23	0.40	0.26	0.18	0.47	0.32	0.23	0.38	0.26	0.18	0.47	0.40	0.12
Pro	CCU	**1.33**	**1.43**	**1.93**	**1.78**	1.35	**3.33**	**1.85**	**1.65**	**2.75**	**1.61**	**1.46**	**1.86**	**2.09**	**1.82**	**2.59**	**1.89**	1.44	**3.11**	**1.84**	**1.45**	**3.16**	**1.79**	**1.41**	**3.53**	**1.72**	**1.49**	**2.30**	**1.67**	**1.78**	**2.06**
	CCC	1.23	1.17	1.19	0.85	1.14	0.22	0.79	0.95	0.50	0.96	1.15	1.29	0.27	0.57	0.00	0.74	1.00	0.44	0.80	1.05	0.21	0.84	1.10	0.24	0.89	1.06	0.48	0.90	0.85	0.77
	CCA	1.30	1.25	0.59	1.33	**1.47**	0.44	1.09	1.12	0.75	1.27	1.27	0.71	1.45	1.51	1.41	1.33	**1.48**	0.22	1.25	1.41	0.63	1.26	1.37	0.24	1.29	1.29	1.09	1.27	1.30	0.90
	CCG	0.14	0.16	0.30	0.04	0.04	0.00	0.27	0.28	0.00	0.16	0.12	0.14	0.18	0.10	0.00	0.04	0.08	0.22	0.10	0.08	0.00	0.11	0.12	0.00	0.10	0.16	0.12	0.16	0.07	0.26
Thr	ACU	**1.38**	1.29	**2.40**	**1.60**	1.32	**2.84**	**1.66**	1.39	**2.53**	1.42	1.26	**2.17**	**1.65**	1.41	**2.07**	**1.62**	1.34	**2.84**	**1.58**	1.31	**2.78**	**1.61**	1.34	**2.92**	1.48	1.36	**2.38**	1.48	1.33	**1.53**
	ACC	1.03	1.08	0.46	0.66	0.71	0.21	0.70	0.77	0.40	0.71	0.77	0.43	0.60	0.60	0.41	0.65	0.71	0.32	0.69	0.73	0.22	0.64	0.71	0.22	0.70	0.64	0.48	0.72	0.70	0.77
	ACA	1.29	**1.33**	0.69	1.52	**1.67**	0.74	1.45	**1.68**	0.80	**1.55**	**1.73**	0.96	1.52	**1.65**	1.38	1.52	**1.67**	0.63	1.48	**1.66**	0.78	1.53	**1.67**	0.76	**1.61**	**1.78**	0.95	**1.50**	**1.68**	1.36
	ACG	0.29	0.29	0.46	0.22	0.30	0.21	0.19	0.16	0.27	0.33	0.25	0.43	0.24	0.33	0.14	0.20	0.28	0.21	0.26	0.30	0.22	0.22	0.28	0.11	0.21	0.22	0.19	0.30	0.29	0.34
Ala	GCU	**1.43**	0.97	**2.27**	**2.16**	**1.66**	**2.87**	**2.02**	**1.39**	**3.31**	**1.81**	**1.40**	**2.70**	**2.42**	**2.41**	**2.40**	**2.04**	**1.71**	**2.63**	**2.12**	**1.77**	**2.44**	**2.14**	**1.69**	**2.84**	**1.98**	**1.68**	**2.47**	**2.48**	**2.11**	**3.09**
	GCC	1.33	**1.95**	0.24	0.71	1.03	0.00	0.88	1.28	0.11	0.92	1.14	0.54	0.28	0.29	0.23	0.82	0.91	0.32	0.73	0.91	0.39	0.74	0.96	0.11	0.53	0.68	0.34	0.74	1.06	0.34
	GCA	0.68	0.77	0.71	1.02	1.26	0.72	1.03	1.28	0.46	1.09	1.30	0.54	1.21	1.24	1.03	1.02	1.26	0.84	1.00	1.20	0.88	1.01	1.24	0.84	1.29	1.42	0.85	0.63	0.72	0.34
	GCG	0.56	0.31	0.78	0.12	0.06	0.41	0.08	0.06	0.11	0.17	0.16	0.22	0.08	0.06	0.34	0.12	0.11	0.21	0.15	0.11	0.29	0.12	0.11	0.21	0.20	0.21	0.34	0.15	0.11	0.23
Tyr	UAU	**1.31**	**1.13**	**1.76**	**1.58**	**1.40**	**1.81**	**1.44**	**1.25**	**1.83**	**1.46**	**1.39**	**1.70**	**1.58**	**1.46**	**1.82**	**1.53**	**1.36**	**1.73**	**1.56**	**1.38**	**1.77**	**1.56**	**1.38**	**1.76**	**1.51**	**1.33**	**1.75**	**1.40**	**1.35**	**1.75**
	UAC	0.69	0.87	0.24	0.42	0.60	0.19	0.56	0.75	0.17	0.54	0.61	0.30	0.42	0.54	0.18	0.47	0.64	0.27	0.44	0.62	0.23	0.44	0.62	0.24	0.49	0.67	0.25	0.60	0.65	0.25
Stop	UAA	**1.11**	**1.45**	**1.07**	**1.17**	**1.76**	0.94	**1.19**	**1.73**	**1.07**	**1.28**	**1.69**	**1.33**	**1.36**	**1.76**	**1.09**	**1.17**	**1.75**	0.98	**1.18**	**1.76**	0.98	**1.18**	**1.75**	**1.00**	**1.21**	**1.81**	**1.57**	**1.27**	**1.71**	**1.36**
	UAG	0.89	0.55	0.93	0.83	0.24	**1.06**	0.81	0.27	0.93	0.72	0.31	0.67	0.64	0.24	0.91	0.83	0.25	**1.02**	0.82	0.24	**1.02**	0.82	0.25	**1.00**	0.79	0.19	0.43	0.73	0.29	0.64
His	CAU	**1.04**	**1.02**	**1.78**	**1.27**	**1.22**	**1.88**	**1.46**	**1.37**	**2.00**	**1.23**	**1.18**	**1.30**	**1.57**	**1.55**	**1.87**	**1.33**	**1.30**	**1.88**	**1.29**	**1.22**	**1.87**	**1.30**	**1.22**	**1.88**	**1.22**	**1.13**	**1.57**	**1.45**	**1.18**	**1.64**
	CAC	0.96	0.98	0.22	0.73	0.78	0.13	0.54	0.63	0.00	0.78	0.82	0.70	0.42	0.45	0.13	0.67	0.70	0.13	0.71	0.78	0.13	0.70	0.78	0.13	0.78	0.87	0.43	0.55	0.82	0.36
Gln	CAA	**1.33**	**1.79**	**1.33**	**1.19**	**1.74**	**1.33**	**1.38**	**1.82**	**1.47**	**1.35**	**1.73**	**1.49**	**1.45**	**1.83**	**1.68**	**1.28**	**1.80**	**1.38**	**1.25**	**1.80**	**1.38**	**1.25**	**1.80**	**1.38**	**1.40**	**1.75**	**1.48**	**1.39**	**1.80**	**1.65**
	CAG	0.67	0.21	0.67	0.81	0.26	0.67	0.62	0.18	0.53	0.65	0.27	0.51	0.55	0.17	0.32	0.72	0.20	0.63	0.75	0.20	0.63	0.75	0.20	0.63	0.60	0.25	0.52	0.61	0.20	0.35
Asn	AAU	**1.22**	**1.14**	**1.74**	**1.44**	**1.36**	**1.68**	**1.45**	**1.33**	**1.75**	**1.42**	**1.36**	**1.55**	**1.66**	**1.57**	**1.80**	**1.46**	**1.40**	**1.63**	**1.46**	**1.39**	**1.68**	**1.42**	**1.37**	**1.63**	**1.41**	**1.39**	**1.43**	**1.39**	**1.36**	**1.59**
	AAC	0.78	0.86	0.26	0.56	0.64	0.32	0.55	0.67	0.25	0.58	0.64	0.45	0.34	0.43	0.20	0.54	0.60	0.38	0.54	0.61	0.32	0.58	0.63	0.38	0.59	0.61	0.57	0.61	0.64	0.41
Lys	AAA	**1.30**	**1.57**	0.78	**1.26**	**1.52**	0.97	**1.37**	**1.65**	0.97	**1.45**	**1.56**	**1.52**	**1.46**	**1.65**	**1.42**	**1.26**	**1.51**	0.97	**1.25**	**1.51**	**1.06**	**1.26**	**1.51**	**1.00**	**1.46**	**1.45**	**1.23**	**1.47**	**1.53**	**1.35**
	AAG	0.70	0.43	**1.22**	0.74	0.48	**1.03**	0.63	0.35	**1.03**	0.55	0.44	0.48	0.54	0.35	0.58	0.74	0.49	**1.03**	0.75	0.49	0.94	0.74	0.49	1.00	0.54	0.55	0.77	0.53	0.47	0.65
Asp	GAU	**1.26**	0.94	**1.42**	**1.44**	**1.29**	**1.70**	**1.54**	**1.40**	**1.79**	**1.42**	0.94	**1.75**	**1.63**	**1.41**	**2.00**	**1.47**	**1.29**	**1.78**	**1.41**	**1.35**	**1.64**	**1.41**	**1.29**	**1.64**	**1.46**	**1.38**	**1.39**	**1.46**	**1.25**	**1.59**
	GAC	0.74	**1.06**	0.58	0.56	0.71	0.30	0.46	0.60	0.21	0.58	**1.06**	0.25	0.37	0.59	0.00	0.53	0.71	0.22	0.59	0.65	0.36	0.59	0.71	0.36	0.54	0.63	0.61	0.54	0.75	0.41
Glu	GAA	**1.22**	**2.00**	**1.00**	**1.43**	**2.00**	**1.62**	**1.38**	**2.00**	**1.28**	**1.37**	**1.85**	**1.38**	**1.34**	**1.93**	**1.56**	**1.39**	**2.00**	**1.48**	**1.44**	**2.00**	**1.62**	**1.44**	**2.00**	**1.60**	**1.56**	**2.00**	**1.76**	**1.29**	**2.00**	**1.19**
	GAG	0.78	0.00	1.00	0.57	0.00	0.38	0.62	0.00	0.72	0.63	0.15	0.62	0.66	0.07	0.44	0.61	0.00	0.52	0.56	0.00	0.38	0.56	0.00	0.40	0.44	0.00	0.24	0.71	0.00	0.81
Cys	UGU	**1.47**	**1.00**	**1.46**	**1.47**	**1.14**	**1.55**	**1.60**	**1.03**	**1.77**	**1.40**	**1.10**	**1.61**	**1.50**	**1.12**	**1.73**	**1.48**	**1.20**	**1.56**	**1.47**	**1.18**	**1.54**	**1.44**	**1.14**	**1.50**	**1.48**	**1.14**	**1.74**	**1.52**	**1.03**	**1.76**
	UGC	0.53	**1.00**	0.54	0.53	0.86	0.45	0.40	0.97	0.23	0.60	0.90	0.39	0.50	0.88	0.27	0.52	0.80	0.44	0.53	0.82	0.46	0.56	0.86	0.50	0.52	0.86	0.26	0.48	0.97	0.24
Trp	UGA	**1.25**	**1.49**	**1.00**	**1.47**	**1.47**	**1.42**	**1.47**	**1.57**	**1.23**	**1.44**	**1.46**	**1.35**	**1.63**	**1.53**	**1.41**	**1.47**	**1.48**	**1.43**	**1.48**	**1.53**	**1.33**	**1.53**	**1.53**	**1.43**	**1.61**	**1.50**	**1.64**	**1.66**	**1.46**	**1.42**
	UGG	0.75	0.51	1.00	0.53	0.53	0.58	0.53	0.43	0.77	0.56	0.54	0.65	0.37	0.47	0.59	0.53	0.52	0.57	0.52	0.47	0.67	0.47	0.47	0.57	0.39	0.50	0.36	0.34	0.54	0.58
Arg	CGU	1.33	0.84	**1.71**	0.82	0.60	**1.87**	1.00	0.60	**1.56**	0.85	0.78	0.80	1.25	1.20	1.67	0.68	0.42	**1.43**	0.62	0.49	**1.33**	0.74	0.50	**1.75**	0.98	0.72	**1.47**	1.19	0.98	1.20
	CGC	0.56	0.90	0.19	0.36	0.80	0.00	0.17	0.68	0.00	0.31	0.52	**1.07**	0.00	0.27	0.00	0.39	0.95	0.00	0.44	0.98	0.22	0.37	0.90	0.00	0.27	0.72	0.21	0.37	0.73	0.20
	CGA	**1.39**	**1.61**	0.95	**2.09**	**1.90**	1.07	**2.00**	**1.87**	1.11	**2.00**	**1.74**	**1.07**	**2.13**	**1.87**	**2.00**	**2.15**	**1.89**	1.14	**2.13**	**1.76**	**1.33**	**2.14**	**1.90**	1.00	**1.87**	**1.85**	1.26	**1.70**	**1.55**	**2.00**
	CGG	0.72	0.65	1.14	0.73	0.70	1.07	0.83	0.85	1.33	0.85	0.96	**1.07**	0.63	0.67	0.33	0.78	0.74	**1.43**	0.80	0.78	1.11	0.74	0.70	1.25	0.89	0.72	1.05	0.74	0.73	0.60
Ser	AGU	**1.15**	**1.17**	**1.78**	**1.15**	**1.25**	**1.55**	**1.07**	**1.17**	1.31	**0.92**	**1.08**	1.17	**1.07**	**1.10**	1.35	**1.19**	**1.28**	**1.61**	**1.17**	**1.29**	**1.52**	**1.17**	**1.29**	**1.55**	**1.10**	**1.30**	**1.53**	**1.04**	**1.20**	1.46
	AGC	0.70	1.11	0.46	0.65	0.91	0.52	0.78	1.15	0.48	0.84	**1.08**	0.84	0.72	0.95	0.65	0.66	0.92	0.46	0.68	0.92	0.58	0.68	0.95	0.52	0.78	0.97	0.53	0.63	0.95	0.35
	AGA	0.94	0.99	1.27	1.01	0.81	1.55	1.00	0.74	**1.91**	**0.92**	0.73	**1.57**	1.00	0.72	**1.65**	1.04	0.84	1.50	1.00	0.82	**1.52**	1.02	0.82	**1.55**	0.88	0.72	1.41	**1.04**	0.80	**1.93**
	AGG	0.86	1.08	1.02	0.61	0.96	0.81	0.76	0.94	0.54	0.63	0.93	0.62	0.70	0.97	0.71	0.63	0.97	0.81	0.68	1.00	0.88	0.64	0.97	0.81	0.64	1.05	0.47	0.63	0.87	0.53
Gly	GGU	0.89	0.40	1.42	1.06	1.00	1.39	1.06	0.83	1.28	0.93	0.98	1.13	1.26	0.74	**1.73**	1.18	1.01	**1.48**	1.14	0.97	**1.41**	1.14	1.01	1.35	1.10	0.92	1.33	**1.53**	1.51	**1.68**
	GGC	0.43	0.53	0.41	0.11	0.15	0.27	0.37	0.54	0.20	0.22	0.24	0.24	0.20	0.32	0.18	0.08	0.15	0.22	0.11	0.15	0.27	0.11	0.15	0.27	0.18	0.05	0.27	0.18	0.24	0.23
	GGA	1.07	**1.89**	0.49	**1.93**	**2.35**	**1.44**	**1.47**	**2.00**	0.99	**1.51**	**2.05**	0.84	**2.01**	**2.79**	1.13	**1.95**	**2.43**	1.37	**1.96**	**2.46**	**1.41**	**1.92**	**2.38**	**1.41**	**2.16**	**2.67**	**1.53**	**1.53**	**1.84**	1.22
	GGG	**1.61**	1.19	**1.68**	0.90	0.50	0.91	1.10	0.63	**1.53**	1.34	0.73	**1.79**	0.53	0.16	0.96	0.79	0.41	0.93	0.79	0.41	0.92	0.84	0.46	0.97	0.56	0.36	0.87	0.76	0.42	0.87

Note: “All” represents the relative synonymous codon usage for all PCGs, “J” represents the relative synonymous codon usage for the PCGs on majority strand, “N” represents the relative synonymous codon usage for the PCGs on minority strand. The bold numbers represent the highest relative synonymous codon usage for each kind of amino acid.

With the exception of first codon positions, G is under-represented compared to C in coding genes on the J-strand (PCGs, tRNAs, CR and intergenic nucleotides), while the G content is higher compared to C in coding genes on the N-strand (PCGs, tRNAs and rRNAs). This base compositional bias is in line with the general trend in the mitogenome toward a lower G content [51].

Base compositional heterogeneity and among-site rate variation (ASRV) are known to affect phylogenetic inference, resulting in the identification of incorrect phylogenetic relationships [52]. The easiest solution is simply to avoid non-stationary genes [53] but most earlier studies used relatively intuitive mitogenome data partitioning schemes, including by gene type (PCG, rRNA and tRNA), by gene, by codon position, by codon and gene, or by the strand on which the coding gene is located [15]. Inevitably, different intuitive partitioning schemes can each result in strong conflicting topologies, especially at deeper phylogenetic levels [25], [54], [55]. Therefore, selection of stationary, reversible compositional homogeneous is vital for reliable phylogenetic inference [52], [56].

Many earlier studies were focused on the A+T content of different genes or regions to investigate the base compositional heterogeneity and among-site rate variation ASRV [57]. For mitogenomes, composition bias of A+T content was verified in most earlier studies; e.g. A+T content was usually over-represented in non-coding regions [58] and the third codon position generally had stronger A+T composition bias compared to the other two codon positions [59] etc.. We asked how variability between PCGs is related to underlying A+T content and its distribution across synonymous and non-synonymous sites.

**Figure 2:**
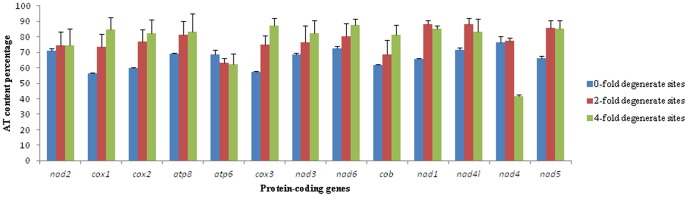
The AT content percentage of 0-fold degenerate sites, 2-fold degenerate sites and 4-fold degenerate sites in each protein-coding gene of mitochondrial genomes of 10 tephritid species, *B. oleae*, *B. tryoni*, *B. philippinensis*, *B. carambolae*, *B. papaya*, *B. dorsalis*, *C. capitata*, *B. minax*, *B. correcta* and *B. curcubitae*. The black line with short line on the top of each bar represents the standard deviation value (SD).

**Figure 3:**
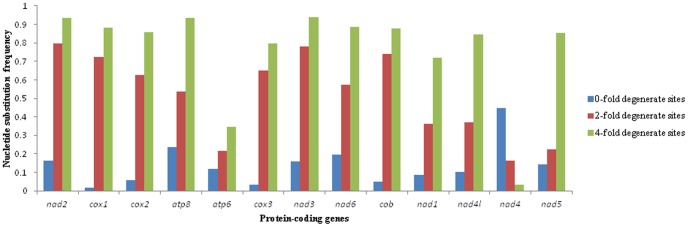
The nucleotide substitution frequency at 0-fold degenerate sites, 2-fold degenerate sites and 4-fold degenerate sites in each protein-coding gene of mitochondrial genomes of 10 tephritid species, *B. oleae*, *B. tryoni*, *B. philippinensis*, *B. carambolae*, *B. papaya*, *B. dorsalis*, *C. capitata*, *B. minax*, *B. correcta* and *B. curcubitae*.

In this study, the A+T content of zero-fold sites (P_0FD_), two-fold (P_2FD_) and four-fold degenerate sites (P_4FD_) was determined for each of the PCGs from ten tephritid species (*B. minax*, *B*. *oleae*, *B. tryoni B. dorsalis B. philippinensis*, *B. carambolae*, *B. papayae*, *B. correcta*, *B. cucurbitae* and *C*. *capitata*) (Fig. 2). Nucleotide substitution frequency was calculated in P_0FD_, P_2FD_ and P_4FD_ for each of the PCGs among five tephritid species (Fig. 3). After analyzing the correlation between A+T content and nucleotide substitution frequency for each of the PCGs, we found a significant positive correlation between A+T content percentage of zero-fold degenerate sites (AT_0F_) and nucleotide substitution frequency at P_0FD_ (*r* = 0.735, *P* = 0.004) as well as between A+T content percentage of four-fold degenerate sites (AT_4F_) and nucleotide substitution frequency at P_4FD_ (*r* = 0.864, *P* = 0.000) (Table 5). Correlation analysis indicated there is a significant positive correlation between AT_0F_ and ASD (*r* = 0.752, *P* = 0.003), ASD and the nucleotide substitution number of zero-fold degenerate sites/the nucleotide substitution number of all degenerate sites (R_0F/all_) (*r* = 0.983, *P* = 0.000), AT_0F_ and R_0F/all_ (*r* = 0.760, *P* = 0.003) (Table 6). Interestingly, the significant positive correlation was observed between AT_4F_ and the nucleotide substitution number of four-fold degenerate sites/the nucleotide substitution number of all degenerate sites (R_4F/all_) (*r* = 0.809, *P* = 0.001); however, there was significant negative correlation between AT_4F_ and ASD (*r* = −0.828, *P* = 0.000), between R_4F/all_ and ASD (*r* = −0.970, *P* = 0.000) (Table 6). On the basis of the above results, we can hypothesize divergence at the amino acid level of less well conserved PCGs is due to higher A+T at P_0FD_ in those genes and/or lower A+T at P_4FD_. On the basis of this result, when we choose which PCGs are used to analyze phylogenic relationships for different evolutionary time scales, the A+T content of P_0FD_ and/or P_4FD_ of PCGs could be useful to judge the homogenesis of PCGs.

**Table 5:** A+T content percentage and nucleotide substitution frequency at 0-fold degenerate sites (P_0FD_), 2-fold degenerate sites (P_2FD_) and 4-fold degenerate sites (P_4FD_) (the number of substitutions per P_0FD_, P_2FD_ and P_4FD_ site)in each PCG of mtgenome of 10 tephritid species, *B. oleae*, *B. tryoni*, *B. philippinensis*, *B. carambolae*, *B. papaya*, *B. dorsalis*, *C. capitata*, *B. minax*, *B. correcta* and *B. curcubitae*.

Protein-coding genes	P_0FD_		P_2FD_		P_4FD_	
	A+T percentage (%)	nucleotide substitution frequency	A+T percentage (%)	nucleotide substitution frequency	A+T percentage (%)	nucleotide substitution frequency
*nad2*	70.96±1.41	0.198	74.61±8.66	0.811	74.33±10.99	1.689
*cox1*	56.53±0.21	0.025	73.31±8.55	0.743	84.93±7.82	1.409
*cox2*	59.71±0.74	0.074	76.92±7.67	0.624	82.47±8.64	1.306
*atp8*	68.92±0.81	0.235	81.54±8.66	0.577	83.33±11.86	1.400
*atp6*	68.76±2.92	0.459	63.36.±2.91	0.227	62.46±6.84	0.590
*cox3*	57.46±0.36	0.034	75.10±5.61	0.655	87.34±4.95	1.266
*nad3*	68.38±1.05	0.192	76.61±10.74	0.831	82.12±8.50	1.485
*nad6*	72.59±1.44	0.265	80.12±8.60	0.655	87.68±4.11	1.326
*cob*	61.62±0.42	0.057	68.66±9.26	0.741	81.14±6.72	1.417
*nad1*	65.54±0.53	0.094	88.33±2.51	0.381	73.72±1.09	1.090
*nad4l*	71.44±1.44	0.107	87.94±4.12	0.397	83.46±8.32	1.154
*nad4*	76.42±3.72	0.783	77.49±1.86	0.172	41.72±0.73	0.004
*nad5*	66.33±1.23	0.172	85.77±4.83	0.313	85.18±5.45	1.234
Correlation coefficient (*r*)	0.735		−0.217		0.864	
Confidence probability (*P*)	0.004<0.01		0.477>0.05		0.000<0.01	

Note: the correlation analysis used Pearson coefficient under two-tailed test of significance.

**Table 6:** The A+T content percentage of 0-fold degenerate sites (AT_0F_), the nucleotide substitution number of 0-fold degenerate sites/the nucleotide substitution number of all degenerate sites (R_0F/all_) and the mean genetic distance based on amino acid sequence (ASD) in each protein-coding gene of mitochondrial genomes of 10 tephritid species, *B. oleae*, *B. tryoni*, *B. philippinensis*, *B. carambolae*, *B. papaya*, *B. dorsalis*, *C. capitata*, *B. minax*, *B. correcta* and *B. curcubitae*, and the correlation coefficient between them (AT_0F_, R_0F/all_ and ASD).

Protein-coding genes	AT_0F_	AT_4F_	R_0F/all_	R_4F/all_	ASD	AT_0F_ & R_0F/all_	AT_0F_ & ASD	R_0F/all_ & ASD	AT_4F_ & R_4F/all_	AT_4F_ & ASD	R_4F/all_ & ASD
*nad2*	70.96±1.41	74.33±10.99	0.290	0.362	0.117	*r* = 0.760, *P* = 0.003<0.01	*r* = 0.752, *P* = 0.003<0.01	*r* = 0.983, *P* = 0.000<0.01	*r* = 0.809, *P* = 0.001<0.01	*r* = −0.828, *P* = 0.000<0.01	*r* = −0.970, *P* = 0.000<0.01
*cox1*	56.53±0.21	84.93±7.82	0.046	0.606	0.014						
*cox2*	59.71±0.74	82.47±8.64	0.148	0.514	0.036						
*atp8*	68.92±0.81	83.33±11.86	0.400	0.350	0.164						
*atp6*	68.76±2.92	62.46±6.84	0.780	0.141	0.280						
*cox3*	57.46±0.36	87.34±4.95	0.068	0.552	0.014						
*nad3*	68.38±1.05	82.12±8.50	0.300	0.350	0.105						
*nad6*	72.59±1.44	87.68±4.11	0.417	0.297	0.168						
*cob*	61.62±0.42	81.14±6.72	0.106	0.497	0.029						
*nad1*	65.54±0.53	73.72±1.09	0.222	0.449	0.052						
*nad4l*	71.44±1.44	83.46±8.32	0.244	0.385	0.053						
*nad4*	76.42±3.72	41.72±0.73	0.936	0.004	0.382						
*nad5*	66.33±1.23	85.18±5.45	0.357	0.431	0.083						

Note: the correlation analysis used Pearson coefficient under two-tailed test of significance.

The A+T content percentage of 4-fold degenerate sites (AT_4F_), the nucleotide substitution number of 0-fold degenerate sites/the nucleotide substitution number of all degenerate sites (R_4F/all_) and the mean genetic distance based on amino acid sequence (ASD) in each protein-coding gene of mitochondrial genomes of 10 tephritid species above, and the correlation coefficient between them (AT_4F_, R_4F/all_ and ASD).

Nucleotide substitution is considered to be a reflection of evolution at the molecular level. Many earlier studies indicated the substitution was directional bias across different genes in the mitogenome [15]. Some researchers have proposed variation of A+T% among taxa is associated with directional mutation pressure and has a phylogenetic component [57], [60], [61]. In this study, with the exception of *nad4*, all PCGs had significantly lower variation of A+T content among the ten tephritid species at P_0FD_ compared to both P_2FD_ and P_4FD_ sites. We observed that, with the exception of *nad4*, P_0FD_ sites had lower nucleotide substitution frequency compared to both P_2FD_ and P_4FD_ sites (Fig. 3). The P_0FD_ of *nad4* had a higher nucleotide substitution frequency (0.783) compared to both P_2FD_ (0.172) and P_4FD_ (0.004), and the R_0F/all_ was 0.936. As a result of functional constraints, the number of nucleotide substitution per non-synonymous site is usually lower than that per synonymous site [62]. In this study, a higher nucleotide substitution frequency at P_0FD_ of *nad4* indicates the non-synonymous nucleotide substitution frequency was higher compared to the synonymous sites for this gene. Higher number of nucleotide substitution per non-synonymous site has been observed at the variable-region genes of immunoglobulins [63] and some genes of the histocompatibility complex [64] but this is the first reported occurrence in the mitogenome.

### 3. Protein-coding genes

With the exception of *cox1* and *nad3*, all protein coding genes start with an ATN codon, with ATG used in *cox2*, *atp6*, *cox3*, *nad4*, *nad4l*, *nad6* and *cob*, ATT in *nad2*, *atp8* and *nad5* and ATA in *nad1*. Genes for *cox1* and *nad3* used TCG and GTC as initiation codons, respectively. The initiation codon for *cox1* was TCG(S) in *B. minax*, which was observed in other Diptera species [54]. GTC being the initiation codon for *nad3* was a new observation in tephritids, but it is common in other insects [65].

With the exception of *nad3*, *nad5* and *nad1*, all PCGs are terminated by complete stop codons: TAG is used for *nad2*, *atp6* and *cob*, TAA is used for *cox2*, *atp8*, *cox3*, *nad4*, *nad4l* and *nad6* and TA is used for *cox1*. The remaining genes, *nad3*, *nad5* and *nad1*, are terminated by incomplete stop codons “T”.

### 4. Transfer RNA genes, ribosomal RNA genes and tRNA-like structure

**Figure 4:**
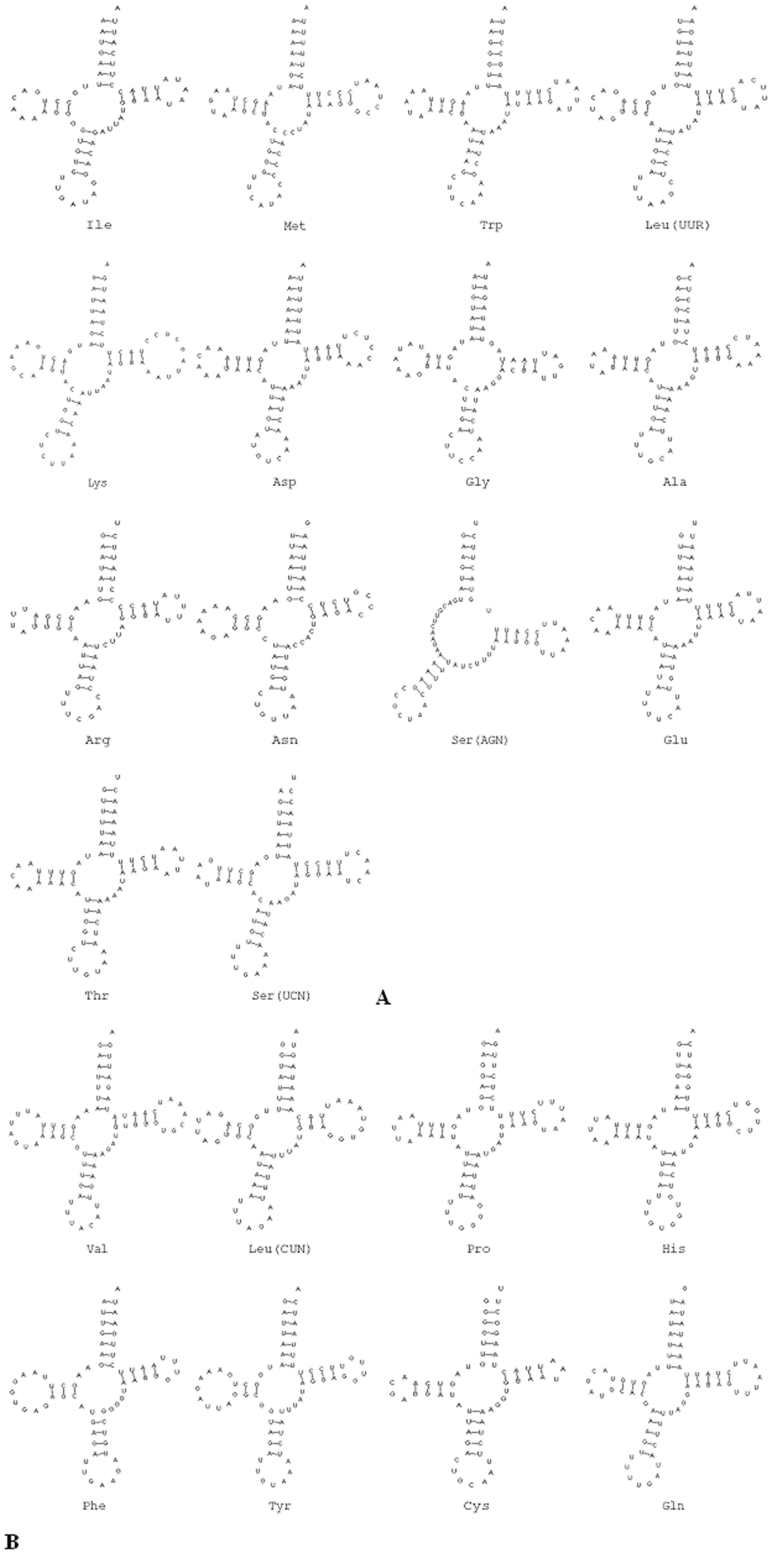
Predicated secondary clover-leaf structures for the 22 tRNA genes of *B. minax*. The tRNAs are labled with abbreviation of their corresponding amino acids below each tRNA gene structure. Arms of tRNAs (clockwise from top) are the amino acid acceptor arm, TΨC arm, the anticodon arm, and dihydrouridine (DHU) arm. (A) J-strand coding tRNAs. (B) N-strand coding tRNAs.

All of 22 tRNA genes typical of metazoan mitogenomes were identified in the *B. minax* mitogenome, and the predicted structures are shown in Fig. 4. All tRNAs display a typical clover-leaf secondary structure, except for *trnS^(AGN)^*, where the DHU arm appears to be replaced by seven unpaired nucleotides, a feature typical of other animal mitochondria [66]. Nuclear magnetic resonance analysis of the tertiary structure of nematode *trnS^(AGN))^* suggested such aberrant tRNA can fit the ribosome by adjusting its structural conformation and function in a way similar to that of usual tRNAs in the ribosome [67].

Like most insect tRNAs, all *B. minax* tRNAs have a length of 7 bp for the anticodon loop, 7 bp for the acceptor stem and 5 bp for anticodon stem. Most of the size variability in the *B. minax* tRNA genes originated from length variation in the DHU arms (loop size 4–9 bp, stem size 3–4 bp) and the TΨC arms (loop size 2–9 bp, stem size 3–5 bp); in addition, *trnA* and *trnH* contained U-U mismatches. *trnS^(UCN)^* encodes an A–C mismatch, *trnH* encodes an A-G mismatch and *trnRtrnR* has a U-C mismatch in the acceptor stem. Additionally, *trnV* contains a U-U mismatch in the TΨC stem.

Anticodon sequences were the same as in *B. dorsalis, B. oleae*, *B. tryoni* and *C. capitata*, which are considered common for other insects, including *Gryllotalpa orientalis* [68], *Philaenus spumarius* [35], *Phthonandria atrilineata* [50] and *Artogeia melete* [36].

On the basis of the sequence similarity of *B. dorsalis,* the two genes coding for the small and the large ribosomal subunits were located in the *B. minax* mitogenome between *trnL^(CUN)^* and *trnV* and between *trnV* and the CR region. The length of *B. minax rrnS* and *rrnL* was 782 bp and 1333 bp, respectively, similar to *B. dorsalis*, *B. oleae* and *C. capitata*.

### 5. Intergenic spacers

In *B. minax*, the two longest intergenic spacers were 42 bp between *trnC* and *trnY* and 28 bp between *trnR* and *trnN*. In *B. dorsalis*, the second longest intergenic spacer was 45 bp between *trnC* and *trnY.* In *B. tryoni*, the second longest intergenic spacer was 33 bp between *trnR* and *trnN* and the third longest intergenic spacer was 30 bp between *trnC* and *trnY*. In *B. oleae,* the longest intergenic spacer was 28 bp between *trnR* and *trnN*. In *B. minax*, however, only a 10 bp intergenic spacer was observed between *trnQ* and *trnM,* which is shorter compared to 66 bp in *B. dorsalis*, 71 bp in *B. tryoni* and 47 bp in *C. capitata* at the same location. Yu et al. [48] reported the 45 bp intergenic spacer located between *trnC* and *trnY* in *B. dorsalis* had a clear counterpart in the CR with the first 33 of 45 bp matching. These counterparts were predicted to form a small internal stem and a long stem structure pairing with the partially complementary sequence in the CR. A similar phenomenon was observed in the *B. tryoni* mitogenome, where both the second longest (33 bp between *trnR* and *trnN*) and the third longest intergenic spacer (30 bp between *trnC* and *trnY*) have clear counterparts (32 out of 33 bases and 25 out of 30 bases, respectively) on the N-strand of the CR. These two intergenic spacers have highly significant similarity and their counterparts were located in the same position of the CR. We asked whether the 42 bp intergenic spacer located between *trnC* and *trnY* in *B. minax* had these features. The first 15/42 bp of the spacer have a clear counterpart in the CR at positions 15,670–15,684. The 42 bp of intergenic spacer was predicted to form two stem-loop secondary structures with 4 bp loops and one with a 3 bp stem and the other with a 4 bp stem. The first 15 of the 42 bp formed one of the two structures; a 4 bp stem with a 4 bp loop and a 3 bp flanking sequence. The counterpart in the CR also formed a long stem structure with the neighboring sequence. Yu et al. [48] compared the 33 bp counterpart in the CR from *B. dorsalis* with the *B. oleae* CR and found 25 of the 33 bp were identical. Surprisingly, of the original 33 bases present in the *B. minax* CR, 23 were identical. Therefore, the results obtained in this study support the hypothesis that the secondary structures of the counterparts in both the intergenic spacer and the CR might have a major role in recombination [48], [69].

The four intergenic spacers in *B. minax*, ISS-1 (18 bp between *trnE* and *trnF*), ISS-2 (14 bp between *nad5* and *tRNA^His^*), ISS-3 (16 bp between *trnS^(UCN)^* and *nad1*) and ISS-4 (10 bp between *nad1* and *trnL^(CUN)^*), were observed to be of similar size in the tephritids *B. dorsalis*, *B. philippinensis*, *B. carambolae*, *B. papayae*, *B. correcta*, *B. cucurbitae*, *B. oleae* and *B. tryoni* (18 bp, 15 bp, 15 bp and 10 bp) and *C. capitata* (18 bp, 18 bp, 16 bp and 10 bp) at the same locations. All intergenic spacers were found at the same locations and have highly significant similarity in percentage identity (71.4–100%; Table 7).

**Table 7:** Locations, length and sequences of four shorter intergenic spacers in 10 tephritid species, *B. oleae*, *B. tryoni*, *B. philippinensis*, *B. carambolae*, *B. papaya*, *B. dorsalis*, *C. capitata*, *B. minax*, *B. correcta* and *B. curcubitae*.

Species	*tRNA^Glu^*- *tRNA^Phe^*		*ND5* - *tRNA^His^*,		*tRNA^Ser(UCN)^*- *ND1*		*ND1* -*tRNA^Leu(CUN)^*	
	Sequence	Size (bp)	Sequence	Size (bp)	Sequence	Size (bp)	Sequence	Size (bp)
*B. minax*	ACTAATTACAATTCACTA	18	TGATATATATTTCA	14	TACTAAATATAATTAC	16	AAAAAACAAG	10
*B. oleae*	ACTAAAATAAATACACTA	18	TGATAAATACTTCAC	15	TACTAAATAAAATTA	15	AAAAAACAAG	10
*B. tryoni*	ACTAAATGGAATACACTA	18	TGACAAATATTTCAC	15	TACTAAATTTTATTA	15	AAAAAACAAG	10
*B. dorsalis*	ACTAAATATAATACACTA	18	TGATAAATATTTCAC	15	TACTAAATTCTATTA	15	AAAAAACAAG	10
*B. philippinensis*	ACTAAATATAATGCACTA	18	TGATAAATATTTCAC	15	TACTAAATTTTATTA	15	AAAAAACAAG	10
*B. carambolae*	ACTAAATATAATACACTA	18	TGATAAATATTTCAC	15	TACTAAATTTTATTA	15	AAAAAACAAG	10
*B. papayae*	ACTAAATATAATACACTA	18	TGATAAATATTTCAC	15	TACTAAATTTTATTA	15	AAAAAACAAG	10
*B. correcta*	ACTAAATTTTATACACTA	18	TGATAAATATTTCAC	15	TACTAAATTATATTA	15	AAAAAACAAG	10
*B. curcubitae*	ACTAAATATAATTCACTA	18	TGATAAATATTTCAC	15	TACTAATTTTTATTA	15	AAAAAACAAG	10
*C. capitata*	ACTAAAAATAATTAACTA	18	TGATAAATAATTTTTCAC	18	TACTAAAATTAATTAA	16	TAAAAACAAG	10

Additionally, all four intergenic spacers have clear counterparts in the CR of the ten tephritid species (data not shown) but these intergenic spacers cannot form the secondary structures (even though some can be predicted to form stem-loop structures with 2–3 bp stems). Some earlier studies focused on longer intergenic spacers with potential secondary structure and tried to find original sequences and structures in the CR [48]. Even among the close tephritid species, however, these longer intergenic spacers had significantly different features, including sequence, length and location. Cameron et al. [70] suggested the possibility that stem-loop structures instead of tRNAs in the 3′ end of PCGs enhance the rearrangement. Two of four small intergenic spaces locate the 3′ end of PCGs without forming stem-loop structures. These results might explain why no rearrangement was found in tephritid species. This is the first report of shorter intergenic spacers with highly conserved sequences and locations among four tephritid species, which should attract more attention to the shorter intergenic spacers, even though the functions of these are not clear.

### 6. CR

The CR has a high A+T content among the mitochondrial genes of both vertebrates and invertebrates, and the initiation of replication is one of the most interesting features of this region [8]. Zhang and Hewitt [71] proposed conserved structural features on the basis of comparison of the CRs of one dipteran and two orthopteran species. These features include: (1) a poly(T) stretch at the 5′ end of the CR; (2) a [TA(A)]*_n_*-like stretch after the poly(T) stretch; (3) a highly conserved stem-loop structure; (4) a stem-loop structure with a highly conserved flanking sequence of a TATA consensus at the 5′ end and a G(A)*_n_*T consensus at the 3′ end; and (5) a G+A-rich sequence downstream of the secondary structure. The *B. minax* CR was found to have three of the five features proposed by Zhang and Hewitt [71].

**Figure 5:**
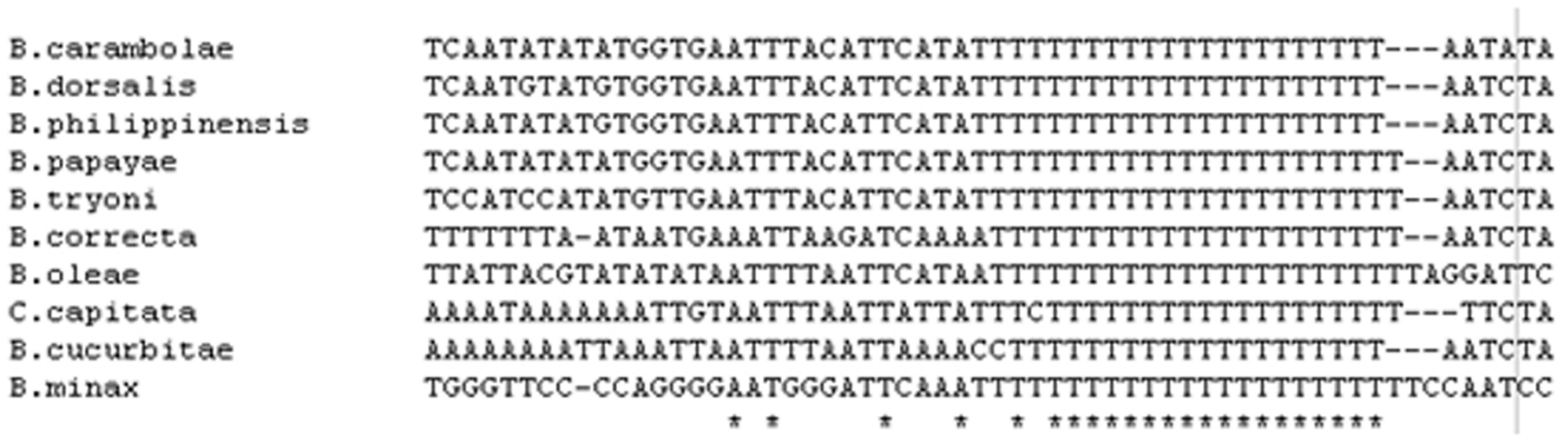
Alignment of the poly-thymidine stretch at the 5′ end of the control region described by Zhang et al. (1997) among 10 tephritid species, *B. oleae*, *B. tryoni*, *B. philippinensis*, *B. carambolae*, *B. papaya*, *B. dorsalis*, *C. capitata*, *B. minax*, *B. correcta* and *B. curcubitae*. The poly-T stretch runs from nucleotide positions from 15974 to 15997 with respect to the *B. minax* mitogenome in the direction of 5′-3′.

The CR from four tephritid species, including *B. minax*, presented a conspicuous poly(T) stretch at the 5′ end. This sequence stretch has been found to be conserved within hymenoptera [49]. Further, the poly(T) stretch has been observed to be followed by a [TA(A)]*_n_*-like stretch (Fig. 5). Our results suggest that this poly(T) region might be involved in the control of transcription and/or replication, or have some other unknown functions [10]. Additionally, a highly conserved G+A-rich sequence block was found in front of the poly(T) stretch among the four tephritid species and these sequences can be predicted to form secondary structures with a stem-loop. The highly conserved G+A-rich sequence with a poly(T) stretch nearby has been found in other dipteran and orthopteran species [71].

In the *B. minax* CR, more than ten sequences have the potential to form stem-loop structures with perfect matches and loops of variable size. In addition, several other stem-loop structures with some mismatch in the stems can be predicted. However, obvious stem-loop structures with conserved flanking sequences were not found in the CR of these ten tephritid species. In addition, The *B. minax* CR does not contain any tRNA-like sequence, but contains two tandem repeats ranging in size from 33 to 45 bp. The sequence TATTAATTTTATTAAA occurred twice and the sequence CCTTTTAAATTTTCC occurred three times. The two repeats were located at positions from 15,325 to 15,357 and from 15,858 to 15,903, respectively. For other tephritid species, we found one tandem repeat in the CR of *B. doraslis*, *B. correcta*, *B. curcubitae* and *C. capitata*, two in *B. philippinensis* and *B. carambolae*, three in *B. oleae* and *B. papaya* but none in *B. tryoni*.
